# Minimal Clinically Important Differences in Measuring Treatment Effects in CIDP: History, Current Use, Limitations, and Prospects

**DOI:** 10.1002/mus.28478

**Published:** 2025-07-25

**Authors:** Yusuf A. Rajabally, Kabir K. Nazeer, Young Gi Min

**Affiliations:** ^1^ Aston Medical School Aston University Birmingham UK; ^2^ Department of Neurology, Inflammatory Neuropathy Clinic University Hospitals Birmingham Birmingham UK; ^3^ Department of Translational Medicine Seoul National University College of Medicine Seoul Republic of Korea

**Keywords:** chronic inflammatory demyelinating polyneuropathy, minimal clinically important differences, outcome measures, treatment, trial

## Abstract

Outcome measures are essential for evaluating treatment effects and disease progression in chronic inflammatory demyelinating polyneuropathy (CIDP). The concept of the minimal clinically important difference (MCID), which represents the smallest change in a measure deemed clinically meaningful, has become increasingly important in CIDP research, and is also gaining interest in clinical practice. This review explores the history of use in clinical trials and observational studies, as well as potential limitations and future perspectives for MCIDs in CIDP. MCID derivation methods include anchor‐based approaches that rely on patient perspectives, and distribution‐based methods that calculate the magnitude of changes exceeding statistical error margins. Both approaches have been used in CIDP, yielding MCID cut‐offs for key scales such as the Inflammatory Neuropathy Cause and Treatment (INCAT) Scale, Overall Neuropathy Limitation Scale (ONLS), Rasch‐built Overall Disability Scale (I‐RODS), grip strength, and the Medical Research Council sum score. Challenges include discrepancies in MCID thresholds, particularly for I‐RODS and strength measures, and variability related to disease severity and subtype. Despite these issues, MCIDs for disability measures such as INCAT, ONLS, and I‐RODS have demonstrated their value through validity and clinical relevance, making them suitable for both research and clinical practice. MCIDs for strength scores, walking tests, sensory scales, and electrophysiological measures lack reliability and direct clinical relevance with regard to the primary concept of clinically meaningful benefit. Future research should focus on optimization of outcome measures, harmonization of MCID derivation methods, and exploration of MCID application with disease‐specific Health Related‐Quality of Life measures for CIDP.

## 1 Introduction

Chronic inflammatory demyelinating polyneuropathy (CIDP) causes functional disability through impairment of motor and sensory functions [1]. A number of strength and disability scales are currently in use in the clinical setting and research to evaluate treatment effects [2]. CIDP is treatable with immunomodulatory therapy. Although multiple earlier trials have demonstrated statistically significant differences between active treatment and placebo arms, the clinical relevance of these differences was not considered. In order for a treatment to be justified, the amplitude of improvement on any scale needs, before other considerations of cost and side‐effect profile, to be clinically meaningful for affected patients. Recent years have seen a greater emphasis on this concept in drug trials, including in CIDP.

Clinical meaningfulness is difficult to define and depends on whether it refers to the patient or physician's perspective, on the disease, its severity at onset, as well as on individual patient characteristics. Disease subtype is equally very influential on the degree of change required to be meaningful, which is highly relevant in CIDP, given its heterogeneous nature. The “Minimal Clinically Important Difference” or “MCID,” was originally defined as the smallest difference in the rating scale under consideration, which patients would perceive as of benefit, and which would lead them to deciding to repeat the intervention, in absence of intolerable side‐effects or excessive cost, if it were their decision [3]. Of importance, the rating scale used needs to have the capability of differentiating such minimal change from random fluctuations observed without therapeutic intervention or changes.

The concept of the MCID allows in principle overcoming the deficiencies of statistically important differences, which fail to consider what matters to the patient as an individual. As such, the MCID allows clinical relevance to be teased out of gains assumed from statistical results and confirms as well as justifies the usefulness of an intervention.

Application of the MCID concept is, in practice, diverse. For CIDP, in which disability measures are commonly used to evaluate treatment effects on physical function, the MCID relates principally to functional benefits experienced by the patient on the utilized scale. However, MCIDs have also been determined in CIDP for non‐disability scales, such as strength measures, sensory scores, or electrophysiology, in which the scale itself is of no direct relevance to the patient, as changes on these measures are only noticed through their correlations with changes in functional ability. Application of the MCID concept may hence arguably be less relevant with such measures.

The clinical relevance of the amplitude of the change will require the use of an “anchor,” which will determine the MCID; therefore, it is directly reliant on patients' perspectives [4]. The anchor is a predetermined level of change in another measure which directly relates to patient well‐being. This anchor is itself dependent upon categorization by the patient of the change, through components of a Health‐Related Quality of Life (HR‐QoL) questionnaire, or through a Patient Global Impression of Change (PGIC) scale. Both “within‐patient” and “between‐patient” differences may be used in this way to ascertain the MCID. Besides this anchor‐based method, distribution‐based methods may alternatively be used to determine the MCID, utilizing the statistical properties of observations of change with an intervention in a patient cohort [5]. With distribution‐based methods, the standard error (SE) of measurement, which measures variations due to measurement error, defines the level below which fluctuations may not be considered as reflective of meaningful change. One SE has been proposed as MCID [6]. However, the standard deviation (SD) which quantifies the spread of data values, has been more widely applied in multiple fields to calculate the MCID, considered to be equal to ½ SD, based on several observations [7], which led to its practical application as a rule of thumb.

Uncertainty remains about optimum methods to determine the MCID. Anchor‐based methods are theoretically preferable, as they rely on the patient perspective [8]. However, they also have possible drawbacks, such as anchor inadequacy due to focus on non‐disease aspects (as may happen when considering patient‐perceived global health status change which may relate to unrelated comorbidities rather than the actual disease) and susceptibility to recall bias for retrospective status recollection. Distribution‐based methods, criticized for not addressing directly the question of clinical relevance, have proved an alternative considered valid in multiple other fields and may be justified given the statistical basis used in multiple aspects of all clinical studies [9]. MCID is also dependent on the direction of change and the variability of illness at baseline [10], which in CIDP, includes disease severity as well as disease subtype. In this regard, the validity of using the same scales and MCID cut‐off for CIDP variants, such as multifocal CIDP in which the deficits are often limited to upper limbs [11] as opposed to typical CIDP, is questionable.

The most recent European Academy of Neurology/Peripheral Nerve Society (EAN/PNS) Guidelines for CIDP from 2021 illustrate the uncertainties regarding the practical application of the MCID concept in the field [12]. A short list of outcome measures with cut‐offs, in some cases variable, was provided as a guide. The list was mainly based on previous MCID utilization in trials and not consistently obtained from previous studies of clinically‐ or research‐derived cut‐offs. Their value for application in the setting of day‐to‐day practice by clinicians, who represent the main target audience of the guidelines, is, as a result, uncertain.

## 2 Ascertainment of MCID for Outcome Measures in CIDP and Application Outside Trials

Multiple studies have been conducted to determine the MCID for CIDP with different scales, over the past 15 years.

The concept of MCID in CIDP is relatively recent and was first considered through a post hoc study [13] of the Intravenous Immunoglobulin (10% caprylate‐chromatography purified) for the treatment of CIDP (ICE) Study [14]. This work evaluated MCID for different scales, determined using one anchor‐based method and calculated through three distribution methods [13]. The anchor‐based method used Question 2 of the Short‐Form 36 (SF‐36) HR‐QoL Questionnaire, in which patients were asked to compare their global health self‐rating at entry and at 24 weeks. The MCID of the evaluated scale was based on the mean score difference self‐rating options of “somewhat better than a year ago” and “about the same as year ago.” The distribution methods to calculate the MCID were (i) ½ SD (ii) SE (iii) effect size. The results demonstrated that the MCID of the primary outcome measure, the adjusted Inflammatory Neuropathy Cause and Treatment (INCAT) Disability Scale, was between 0.60 and 0.72. These results, therefore, provided retrospective justification for use of the applicable cut‐off of 1‐point on the INCAT scale to define improvement in the ICE Trial and brings support for its continuing use in CIDP trials to this day. MCID discrepancies between ascertainment methods were, on the other hand, found for grip strength (14 kPa with the anchor‐based method vs. 8 kPa using ½ SD or SE), and MRC sum score (4 points with the anchor‐based method or ½ SD vs. 2 points using SE). In addition, this analysis also determined the MCIDs for INCAT sensory sum score, electrophysiological parameters, the Rotterdam Handicap Scale, and the SF‐36 HR‐QoL scores. As acknowledged by the authors, this first study of MCID in CIDP did not consider baseline disability, nor did it separate disease subtypes. The nonlinearity of the adjusted INCAT scale was also acknowledged as an issue, as a single MCID may not be applicable at all points on the scale. The authors proposed, for future CIDP research, the approach of “combined MCIDs,” as advocated in other fields, combining one anchor‐based and one distribution‐based method.

Draak et al. subsequently reported on a comparative study of responsiveness of the inflammatory Rasch‐built Overall Disability Scale (I‐RODS), the INCAT, and the ONLS in subjects with CIDP, as well as Guillain–Barré syndrome (GBS) and IgM monoclonal gammopathy of uncertain significance (MGUS) [15]. The comparison was done at the individual person level, using individual SE after Rasch transformation of all three scales. In order to define response on each scale, the MCID was computed by dividing the individual score changes by their corresponding SE. The result, defined as “MCID‐SE,” was used to define responder status, and clinically important improvement required an “MCID‐SE” ≥ 1.96. This cut‐off relates to the requirement for the post‐intervention measurement to be beyond the 95% CI of the pre‐intervention measurement (*T*1 + 1.96 × T1SE; *T*1 representing the pre‐intervention measurement), to be of significance [16].

Vanhoutte et al. described the findings of a further study of comparative responsiveness of the I‐RODS with Rasch‐transformed MRC sum score and Rasch‐transformed INCAT sensory sum score, in patients with CIDP and GBS [17]. Also challenging the concept of single SE values across the range of scales, they determined individual SE for each patient and determined the MCID‐SE in each case. The SE had largest amplitudes at both ends of all evaluated scales and was at its lowest in the middle, in a “U‐shaped” graphical pattern. Clinically meaningful improvement in a given subject was attained if their MCID‐SE ≥ 1.96, relating, as per above, to the requirement to exceed 95% CI of pretreatment performance [16].

In a study comparing two types of devices to evaluate grip strength in inflammatory neuropathies, including CIDP, GBS, IgM MGUSP, and multifocal motor neuropathy (MMN), Draak et al. evaluated responsiveness through the MCID, with an anchor‐based method using question 2 of the SF‐36 HR‐QoL questionnaire and a distribution method using ½ SD [18]. The MCID cut‐offs obtained were, of note, markedly dissimilar with the two methods (6 and 10.5 kg for the Jamar dynamometer, and 6 and 10 kPa for the Martin vigorimeter, for the anchor‐based and distribution methods, respectively).

Draak et al. proposed in 2018 a new, Rasch‐constructed, disease‐specific HR‐QoL measure for inflammatory neuropathy, the IN‐QoL [19]. This was based on data from six QoL questionnaires, ultimately selecting 73 items (consisting of 55 of the “mental” subset, and 18 of the “functional” subset) from an original 184 items, through exclusions resulting from item fit and threshold analysis, consideration of item intercorrelation, and group factor ascertainment. In this study, responsiveness was determined through the MCID obtained for each subject, using individual SE. In subjects with CIDP, this method showed poor responsiveness for the “functional” subset of the scale and comparable responsiveness for the “mental” subset and the global scale to that of the EQ‐VAS, a non‐disease‐specific HR‐QoL measure. These results necessarily raise concerns about the usefulness of the IN‐QoL as a disease‐specific HR‐QoL measure.

In a study of the value of the 6‐minute walk test in CIDP (6MWT), Spina et al. evaluated 42 patients pre‐ and at 2 months post‐treatment [20]. The MCID for the 6MWT was determined using an anchor‐based method considering the mean value difference in walked metres between “stable” and “slightly improved” groups. Their distribution‐based method was performed through calculation of the minimal detectable change (MDC) and effect size. The final MCID was considered as the mean of the anchor‐based value, MDC, and result for small effect size (0.2 × SD). This was of 20 m. Of note, however, the more straightforward ½ SD method produced a comparable result of 21.3 m. In relation to the issue of possible MCID variation dependent on pretreatment status, no association was found between the derived MCID and baseline 6MWT.

We performed, in our unit, a retrospective study of the comparative value of three outcome measures for CIDP: the Medical Research Council (MRC) sum score (MRCSS) in eight paired muscle groups, the Overall Neuropathy Limitation Scale (ONLS) [21], a scale derived from and closely related to the INCAT, and the raw I‐RODS [22]. In order to determine respective scale sensitivities to detect clinically meaningful change in a cohort of 35 subjects, we derived the MCID exclusively through a distribution method using ½ SD. The applicable MCID cut‐offs found for the MRCSS and ONLS were similar to those derived previously for MRCSS and INCAT through anchor‐based methods (4 and 1, respectively) and the MCID cut‐off was 4 points for the raw I‐RODS.

We subsequently conducted a larger retrospective study of 72 subjects with CIDP, with self‐determined treatment response [23]. We aimed to ascertain comparative scale sensitivities, as well as combined sensitivities, through MCID cut‐offs derived in this cohort for four scales, after ensuring this value was greater than the MDC_95_ (Minimum Detectable Change with 95% confidence intervals). This was of importance as the MDC, which is the smallest measurable difference on the scale greater than the measurement error, needs to be smaller than the MCID for the latter to be applicable. We determined MCID through a distribution method using one‐half SD for each of the four scales, which included the MRCSS, the ONLS, the centile I‐RODS, and grip strength. The MCID for the MRCSS was smaller than the MDC_95_, indicating its inadequacy in detecting change. The applicable MCID cut‐off for the ONLS was 1, confirmatory of our previous analysis. The applicable MCID cut‐off for the centile I‐RODS was 8. As for grip strength, the applicable MCID cut‐off was 4 kg, this contrasting with that of 10.5 kg found by Draak et al. [18], using the same distribution method.

GRIPPER, a prospective observational study, explored treatment‐related fluctuations with IVIg infusions in 25 subjects with CIDP [24]. A cut‐off of 10% relative change from baseline over 3 consecutive days was preselected to separate random changes or “noise,” from clinically meaningful change. This corresponded to two SDs of the mean daily grip strength fluctuation. The results demonstrated that, with 3‐day averaged recordings, consecutive averaged day readings exceeding 10% change occurred in only 1.6% for the dominant hand. They concluded that this supported the notion of a 10% relative change in grip strength over 3 consecutive days' averaged measurements as clinically meaningful. In effect, therefore, the results suggested that a 10% cut‐off could be an adequate MCID.

Keh et al. attempted to predict long‐term trends of outcome measures during periods deemed clinically stable with IVIg treatment, in subjects with CIDP and MMN, using latent class modeling [25]. They found that intraindividual variations exceeded the MCID for I‐RODS, MRCSS and grip strength, in 44.2%, 43.5%, and 10.6% of subjects with CIDP, respectively. Importantly, however, their selected I‐RODS MCID was 4 centile points (not previously derived) and 2 points for the MRCSS (derived in one study through SE, but diverging with other methods [13]), whereas that for grip strength was of 8 kPa (derived through ½ SD and SE, but diverging from the anchor‐based approach). Through latent class modeling, they identified deteriorators and improvers, with censoring events being “an increase of IVIg dose or clinical relapse as judged by the treating neurologist.” They found that the best model for deteriorator classification was contiguous sub‐MCID decline in more than one outcome measure (sensitivity: 74%, specificity: 59% for CIDP) which they proposed as superior to an MCID‐exceeding change in a single measure. The inadequacy of the MCID cut‐offs applied for both I‐RODS and MRCSS, and the use of subjective clinical judgments as censoring events, cast doubt on the results and conclusions.

Llauradó et al. conducted a study of 20 subjects with CIDP to determine the MCID of gait evaluations including time up and go (TUG), 10‐metre walk test (10MWT) and 30‐s chair stand (30SCS) [26]. With receiver operating characteristic (ROC) curves and using clinical status judged through attainment of MCIDs of 4 centile points for I‐RODS, 1 point for INCAT, 4 points for MRCSS, or 8 kPa for grip strength, optimally sensitive and specific cut‐offs for each gait scale were ascertained. Besides the optimal cut‐offs reported being only 1 s for TUG and 10MWT and 1 repetition for 30SCS, and therefore intuitively subject to errors of measurement, the reliance on other scales with debatable MCIDs as anchors raises methodological issues that may have impacted the results.

## 3 Application of the Concept of MCID in CIDP Therapeutic Trials

The MCID was considered in many, but not all, drug trials performed in CIDP since 2010.

Details of study publications of the most important trials using MCID are provided in Table 1. A breakdown of the frequency of use of different MCIDs is provided in Table 2.

**TABLE 1:** Examples of MCID applications and their purposes in clinical trials and their post hoc analyses for CIDP.

Author, study	Trial design	Drug	Used outcome measures and cut‐off	Used to define	Endpoint
Nobile‐Orazio et al. [27]	Randomized, double‐blind, placebo‐controlled	IVIg (*n* = 24) vs. IVMP (*n* = 21)	ONLS (or mRS), 1‐point	Improvement	Secondary
Léger et al. [28] “PRIMA”	Single‐arm, open‐label	IVIg (*n* = 28)	aINCAT: 1‐point	Improvement	Primary
			MRCSS (0–80): 3‐point		Secondary
Merkies et al. [29]	PRIMA post‐hoc analysis		INCAT: 1‐point MRCSS (0–80): 4‐point Grip strength: 8 kPa		Post‐hoc analysis
van Schaik et al. [30] “PATH”	Randomized, double‐blind, placebo‐controlled	High‐dose SCIg (*n* = 58) vs. low‐dose SCIg (*n* = 57) vs. placebo (*n* = 57)	aINCAT: 1‐point I‐RODS: 4‐centile Grip strength: 8 kPa	IVIg dependency	Run‐in period
			aINCAT: 1‐point	Relapse	Primary
Merkies et al. [31]	PATH post‐hoc analysis		I‐RODS: (i) 4‐raw, (ii) 8‐centile, (iii) 1.96 SE		Post‐hoc analysis
Merkies et al. [31]	PRIMA & PATH combined analysis	IVIg (*n* = 235; PRIMA, *n* = 28; PATH, *n* = 207)	aINCAT: 1‐point MRCSS (0–80): 3‐point Grip strength: 8 kPa	Improvement	Post hoc analysis
Mielke et al. [31]	PATH post‐hoc analysis	IVIG (*n* = 207)	aINCAT: 1‐point MRCSS (0–80): 3‐point Grip strength: 8 kPa	Improvement	Post‐hoc analysis
Hughes et al. [31] “FORCIDP”	Randomized, double‐blind, placebo‐controlled	Fingolimod (*n* = 54) vs. placebo (*n* = 52)	aINCAT: 1‐point	Worsening	Primary
Nobile‐Orazio et al. [32] “PRISM”	Single‐arm, open‐label	IVIg (*n* = 44: 22 Ig‐naïve, 21 Ig‐pretreated)	aINCAT: 1‐point	Improvement	Primary
Rajabally et al. [33]	PRISM post‐hoc analysis		I‐RODS: 4‐raw MRCSS (0–80): 4‐point Grip strength: 8 kPa		Post‐hoc analysis
Cornblath et al. [34] “ProCID”	Randomized, double‐blind, placebo‐controlled	IVIg 2 g/kg (*n* = 36) vs. IVIg 1 g/kg (*n* = 69) vs. IVIg 0.5 g/kg (*n* = 34)	aINCAT: 1‐point I‐RODS: 1.96 SE Grip strength: 8 kPa	IVIg dependency	Run‐in period
			aINCAT: 1‐point	Improvement	Primary
			Grip strength: 8 kPa I‐RODS: 1.96 SE MRCSS (0–80): N/A		Secondary
Bus et al. [35] “OPTIC”	Randomized, double‐blind, placebo‐controlled	IVIg plus IVMP vs. IVIg plus placebo (ongoing)	aINCAT: 1‐point I‐RODS: 1.96 SE	Remission	Primary
Adrichem et al. [36] “IOC”	Randomized, double‐blind, IVIg‐controlled, non‐inferiority	IVIg withdrawal (*n* = 29) vs. IVIg continuation (*n* = 31)	I‐RODS: 0.65 logit or 1.96 SE^a^	Relapse	Primary
			I‐RODS: 1.96 SE grip strength: 8 kPa	Re‐stabilization	Restablisation phase
			I‐RODS: 1.96 SE	Stable disease	Extension phase
van Veen et al. [37]	IOC post‐hoc analysis		I‐RODS: (i) 4‐centile, (ii) 1.96 SE Grip strength: (i) 8 kPa, (ii) 14 kPa MRCSS (0–60): (i) 2, (ii) 4 point^b^ Composite 1: I‐RODS 4‐centile or grip strength 8 kPa or MRCSS 2‐point Composite 2: I‐RODS 4‐centile or grip strength 14 kPa or MRCSS 2‐point Composite 3: I‐RODS 4‐centile or MRCSS 2‐point Composite 4: I‐RODS 1.96 SE or MRCSS 2‐point	Worsening	Post‐hoc analysis
Bril et al. [38] “ADVANCE‐CIDP1”	Randomized, double‐blind, placebo‐controlled	Facilitated SCIg (*n* = 62) vs. placebo (*n* = 70)	aINCAT: 1‐point	Relapse	Primary
			Composite: aINCAT 1‐point or I‐RODS 4‐raw or Grip strength: 8 kPa	Relapse	Secondary
Hadden et al. [39] “ADVANCE‐CIDP3”	Open‐label extension of ADVANCE‐CIDP1	Facilitated SCIg (*n* = 85)	aINCAT: 1‐point	Relapse	Exploratory endpoint
Querol et al. [40]	Randomized, double‐blind, placebo‐controlled and open‐label extension	Rozanolixizumab (*n* = 16) vs. placebo (*n* = 17)	aINCAT: 1‐point I‐RODS: 1.96 SE Grip strength: 14 kPa Composite: aINCAT 1‐point or I‐RODS 1.96 SE or Grip strength 14 kPa	Relapse	Secondary
Allen et al. [41] “ADHERE”	Randomized‐withdrawal, double‐blind, placebo‐controlled	Efgartigimod (*n* = 322) (Stage A) Efgartigimod (*n* = 111) vs. placebo (*n* = 110) (Stage B)	aINCAT: 1‐point I‐RODS: 4‐centile Grip strength: 8 kPa	Active disease	Run‐in period
			aINCAT: 1‐point I‐RODS: 4‐centile Grip strength: 8 kPa	Improvement	Primary (Stage A)
			aINCAT: 1‐point	Improvement	Primary (Stage B)

Abbreviations: aINCAT, Adjusted Inflammatory Neuropathy Cause and Treatment; CIDP, chronic inflammatory demyelinating polyradiculoneuropathy; I‐RODS, Inflammatory Rasch‐built Overall Disability Scale; IVIg, intravenous immunoglobulin; IVMP, intravenous methylprednisolone; MCID, minimal clinically important difference; MRCSS, Medial Research Council sum score; mRS, Modified Rankin Scale; ONLS, Overall Neuropathy Limitation Scale; SCIg, subcutaneous immunoglobulin; SE, standard error.

^a^ A change of 0.65 or greater was defined as a relapse initially, and sample size estimation was conducted based on this threshold. However, a subsequent protocol amendment introduced a new criterion based on 1.96 SE.

**TABLE 2:** The usage of each outcome measure and its cut‐off in clinical trials and their post hoc analyses for CIDP.

Outcome measure	Number of times used	Cut‐off	References
aINCAT	13	1‐point	[28, 29, 30, 31, 32, 33, 34, 36, 37, 40, 41, 42, 43]
ONLS	1	1‐point	[27]
MRCSS (0–80)	3	3‐point	[28, 31, 32]
	2	4‐point	[29, 33]
	1	NA	[34]
MRCSS (0–60)	1	2‐ or 4‐points	[37]
I‐RODS	2	4‐centile	[30, 41]
	2	4‐raw	[33, 38]
	4	1.96 SE	[34, 35, 36, 40]^a^
	1	4‐raw, 8‐centile, or 1.96 SE	[31]
	1	4‐centile or 1.96 SE	[37]
Grip strength	9	8 kPa	[29, 30, 31, 32, 35, 36, 38, 40, 43]
	1	8 or 14 kPa	[37]
	1	14 kPa	[40]

Abbreviations: aINCAT, adjusted inflammatory neuropathy cause and treatment; I‐RODS, Inflammatory Rasch‐built Overall Disability Scale; MRCSS, Medial Research Council sum score; SE, standard error.

^a^ Adricem et al.—A change of 0.65 or greater was defined as a relapse initially. However, a subsequent protocol amendment introduced a new cut‐off, 1.96 SE.

Of a total of 18 CIDP trial analyses and post hoc analyses [27, 28, 29, 30, 31, 32, 33, 34, 35, 36, 37, 38, 39, 40, 41, 42, 43, 44] that considered the MCID, 13 (72.2%) used the adjusted INCAT, in all cases with a cut‐off of one point in keeping with the originally derived clinically applicable result. The ONLS scale, closely related to the INCAT, was used in one study (5.6%).

Ten studies (55.6%) used the I‐RODS, some with multiple MCIDs, of which 3/10 set the cut‐off at 4 centile points and another 3/10 at 4 raw points. Six of 10 analyses used individualized MCID‐SE ≥ 1.96. The PRIMA trial derived an MCID of 4 raw points within the study cohort itself by anchor‐based and distribution methods. A post hoc study of the IOC trial demonstrated that MCID‐SE ≤ −1.96 offered 98% specificity for clinical deterioration, whereas a drop of 4 centile points was only 77% specific. A subsequent post hoc analysis of PRIMA, otherwise, demonstrated equivalence of an I‐RODS MCID of 4 raw points, individualized MCID‐SE ≥ 1.96, as well as of 8 centile points, as previously derived in our retrospective study [23]. Of note, no justification for use of an I‐RODS MCID of 4 centile points could be found through previous or per‐study derivation, including in the recent ADHERE trial where it was nevertheless used.

Eleven studies (61.1%) used grip strength through Martin vigorimeter readings with an MCID cut‐off exclusively of 8 kPa in 9/11, as previously derived using ½ SD and SE [13]. One study used cut‐offs of 8 kPa and of 14 kPa, the latter previously derived through anchor‐based methods [13]. Another more recent trial considered a single cut‐off of 14 kPa. The post hoc study of the IOC Trial studied the specificity of grip strength MCID cut‐offs versus the absence of decline defined by the treatment anchor. This was low (58% using 8 kPa and 76% using 14 kPa), suggesting inadequacy of both and especially of the lower cut‐off, due to changes in grip strength of such magnitude occurring frequently without treatment modifications due to patient‐ or physician‐identified variations [37]. Of note, although this study was published in 2022, only one of three subsequently published trials opted for an MCID of 14 kPa instead of 8 kPa.

The MRCSS was used with a pre‐established MCID in 6/18 (33.3%) studies. A cut‐off of 3 points was used in three trials and of 4 points in another two trials. In all these trials, the maximum MRCSS was 80 (eight paired muscle groups). One further study considered MCIDs of 2 and of 4 points for a maximum MRCSS of 60 (six paired muscle groups) [37]. In that study, a high specificity of 99% was found for an MCID of 4 points versus the absence of decline as per the treatment anchor.

The post hoc study of the IOC Trial raised some interesting points [37]. This analysis concluded that there was substantial variability for all scales in patients without deterioration as defined by the treatment anchor, describing most MCIDs as located within the limits of variability in stable patients. As such, these findings may cast doubt as to the validity of the MCID concept itself. However, in this study, the anchor for deterioration described as the “treatment anchor,” was defined as either (i) MCID‐SE ≤ −1.96 on the I‐RODS, or (ii) changes judged as relevant by the patient or the clinician. Of note, the latter condition resulted in a therapeutic decision in 50% of cases. That the introduction of such a subjective definition for the treatment anchor affected the findings is likely, as is the acknowledgement by the authors that I‐RODS changes may have themselves also impacted physicians' decisions to treat. The calculation of the sensitivity and specificity of an MCID‐SE ≤ −1.96, alone or in combination, using an anchor partly based on this same MCID, appears otherwise a circular argument. Even with the inclusion of this subjective bias, the detailed results of the study showed high MCID specificity with MCID‐SE ≤ −1.96 for the I‐RODS and of a 4‐point drop for the MRCSS (/60), somewhat contrary to the authors' conclusion that “clinically important deterioration cannot be distinguished from variability over time with currently used MCIDs on the individual level.” Finally, the high sensitivities of some of evaluated MCIDs to correctly identify deterioration, although desirable in the clinical setting, may be challenged as unreliable, also due the study's use of a subjective anchor, itself possibly additionally influenced by the effect of score changes on treating physicians who were unblinded to them.

## 4 MCID for CIDP: Prospects

The concept of MCID in inflammatory neuropathy at large, and CIDP in particular, is relatively recent. As in other fields, differences in MCID derivation methods and variable weight given to disease severity and achieved end‐point have led to differences in practice. Use of arbitrary and unestablished cut‐offs has also unfortunately persisted in absence of consensus. As the most obvious example, despite the inadequacy of a ≥ 4‐point change on the centile I‐RODS score considering the centile point distribution across the scale spectrum and the availability of published alternative distribution‐based values and individualized methods, this cut‐off has continued to be used in recent trials of new agents [41]. Similarly, the likely inadequately low MCID cut‐off for the MRCSS of 2 points, based on historical application and re‐enforced by one discrepant finding through statistical derivation, remains in use [12].

Muscle strength correlations with disability scores are well‐demonstrated in CIDP in multiple studies, although as opposed to disability measures, they do not directly evaluate a functionally relevant change. Several other factors besides strength, including sensory dysfunction, fatigue, mood, but also age, gender, and comorbidities, also have important roles in disability. Furthermore, unequal width of response options has been shown to result in disordered thresholds, impacting raters' ability to distinguish between different MRC scores [42]. As such, each MRC score is subject to this bias, and the effect is compounded through the summation of scores from multiple muscle groups. In addition, we found the MDC to be greater than the MCID for the MRCSS from eight paired muscle groups in one study, suggesting that the use of the MCID may not be valid for that scale [23]. Another issue in relation to the MRCSS is the variability between studies in the number of muscle groups evaluated (6–8, or), which likely impacts the MCID derived. As for grip strength, notable differences have been described between derived MCIDs, using both anchor‐based and distribution‐based methods [18]. The reasons for these discrepant findings are probably multiple, including patient population characteristics and the spectrum of disease subtype and severity.

CIDP disease‐specific MCIDs have been derived through one study for the 6MWT [20]. MCIDs for the more commonly used 10MWT and TUG have recently been proposed in CIDP but with sample size and methodological limitations making their practical applicability uncertain [26]. In addition, despite reproducibility described in other disorders, fatigue, common in CIDP, has been shown to impact upon timed walking tests [43]. Furthermore, similar to strength scores, timed walking tests are not directly relevant to patient well‐being, in contrast to the recovered/improved capability to do a specific common task, as ascertained in a disability measure.

Disability measures are now widely accepted as the optimal available method for evaluation of treatment effects in CIDP [12]. Hence, ascertainment of adequate MCIDs for disability measures has become essential for research and is gaining interest and importance in clinical practice. For the INCAT and subsequently also for the ONLS scale, the derived applicable cut‐off of 1 point is now commonly used, and no other MCID cut‐offs have been proposed. In contrast, for the I‐RODS, heterogeneity remains. Although theoretically preferable, individualized MCIDs using subject‐specific SE are not straightforward in their application as they require calculation through several complex formulae. Standardized MCIDs for the raw I‐RODS, as well as the centile I‐RODS, corresponding to 4 and 8 points, respectively, are easy to apply and have been derived by a few studies, to date. Importantly, a comparative post hoc analysis of an SCIg trial demonstrated that these two standardized MCID cut‐offs offered comparable sensitivity in detecting change to that of the individualized cut‐offs [31]. However, when assuming different baseline levels of disability for a patient, the magnitude of a meaningful change suggested by these 3 cut‐offs is clearly discrepant. Whereas centile score change requirements are equivalent in the middle of the spectrum of severity, major differences are observed with the three different methods in situations of greatest and least severity, in which the MCID thresholds can be as far apart as 11 centile points. Further consideration is, therefore, needed on how best to define the MCID for subjects with very severe or very mild disabilities.

In recent years, as illustrated by this review, considerable complexity has been brought to the discussion through dedicated studies on the subject, as well as to its application. Suggestions of inadequacy of most available MCIDs, including individualized cut‐offs, have been made, particularly in relation to the MCIDs being within the limits of variability in subjects described as stable [25, 37]. However, the designs of these analyses relied on subjective patients' and physicians' opinions and decisions as anchors. Furthermore, although in one study, the 95% CI of the limits of variability in the so‐called stable subjects exceeded the MCIDs, the specificity was 98% for the individualized I‐RODS cut‐off; that is, only 2% of visits without clinical deterioration, as per the anchor, demonstrated changes in excess of the MCID [37]. It is otherwise also noteworthy that two studies that found the overall MCID to be inadequate chose inadequate cut‐offs in the first place, consisting of 4 points for the centile I‐RODS for both, and 2 points for the MRCSS in one [25], resulting in what may be considered unsurprising conclusions. In summary, it may be argued that the evidence of inadequacy of MCIDs for disability measures, derived through accepted methods, is currently unconvincing. Consequently, although requiring further research, the MCID concept should not be dismissed as inapplicable.

The use of HR‐QoL measures has remained marginal in CIDP research, to date [44]. Neither the Chronic Acquired Polyneuropathy Patient‐Acquired Index (CAP‐PRI) Scale [45, 46], nor the IN‐QoL [19], both disease specific, have been used in recent major studies. However, the value of these scales, as well as generic HR‐QoL scales, resides in allowing a holistic approach to the health status of patients, and enabling identification of change with intervention, in specific areas of QoL. In their original study [13], Merkies et al. reported a higher proportion of responders in the treated group versus the placebo group, using the anchor‐based MCID for the Physical Component Score (PCS), but not the Mental Component Score (MCS) of the SF‐36. Results with use of distribution‐based MCIDs were not provided, nor whether statistical significance was reached for the PCS. Considering the close and obvious relationship between the MCID concept and HR‐QoL, illustrated by the use of components from the SF‐36 itself as anchor in this study and others, further research is desirable into the application of MCID to HR‐QoL measures. These correlate well with disability scores while also potentially capturing otherwise undetected benefits of intervention [47], including drug administration modalities [48], of particular relevance with the advent of new agents.

## 5 Conclusions

Outcome measures for CIDP all have numerous limitations, which, however, do not put in doubt their usefulness as currently best available tools for disease and treatment monitoring. Similarly, the value of the MCID concept, despite limitations, appears clear. The findings of this review bring support to wider application of the concept in both research and clinical practice, specifically for disability measures, in particular the INCAT, ONLS, and I‐RODS, for which little variability has been reported for respective cut‐offs, irrespective of derivation methods used and populations studied. Suggestions of inadequacy of derived MCIDs for the above‐mentioned disability scales appear, so far, unconvincing. With regard to strength measures, there exist major discrepancies between studies for derived MCIDs, large fluctuations exceeding published MCIDs in the absence of disability score changes, and uncertainty regarding the clinical meaningfulness of fluctuations. Together with the inherent limitations of these scales, these findings raise doubts regarding the appropriateness of use of their available MCIDs. Similar remarks may be made about timed walking tests, particularly in the presence of limited data. There is currently no support for the use of sensory scores and electrophysiological measures for CIDP monitoring, for which the use of MCIDs appears of little relevance. Finally, as the concept of MCID relates primarily to patient‐perceived meaningful benefit, its application to HR‐QoL measures appears highly appropriate and desirable, as is a greater emphasis on the use of such measures in research and clinical management of subjects with CIDP.

## Author Contributions

**Yusuf A. Rajabally:** conceptualization, data curation, formal analysis, investigation, methodology, project administration, supervision, validation, writing – original draft. **Kabir K. Nazeer:** formal analysis, investigation, project administration. **Young Gi Min:** conceptualization, formal analysis, investigation, methodology, project administration, writing – original draft.

## Ethics Statement

We confirm that we have read the Journal's position on issues involved in ethical publication and affirm that this report is consistent with those guidelines.

## Conflicts of Interest

Y.A.R. has received speaker/consultancy honoraria from Argenx, Sanofi, Grifols, Dianthus, Takeda, Johnson & Johnson, LFB, and Polyneuron; has received educational sponsorships from LFB and CSL Behring; and has obtained research grants from LFB. K.K.N. has received a research grant from LFB. The other author declares no conflicts of interest.

## Data Availability

The authors have nothing to report.
